# On the Power and the Systematic Biases of the Detection of Chromosomal Inversions by Paired-End Genome Sequencing

**DOI:** 10.1371/journal.pone.0061292

**Published:** 2013-04-23

**Authors:** José Ignacio Lucas Lledó, Mario Cáceres

**Affiliations:** 1 Institut de Biotecnologia i de Biomedicina, Universitat Autònoma de Barcelona, Bellaterra, Barcelona, Spain; 2 Institució Catalana de Recerca i Estudis Avançats, Barcelona, Spain; Auburn University, United States of America

## Abstract

One of the most used techniques to study structural variation at a genome level is paired-end mapping (PEM). PEM has the advantage of being able to detect balanced events, such as inversions and translocations. However, inversions are still quite difficult to predict reliably, especially from high-throughput sequencing data. We simulated realistic PEM experiments with different combinations of read and library fragment lengths, including sequencing errors and meaningful base-qualities, to quantify and track down the origin of false positives and negatives along sequencing, mapping, and downstream analysis. We show that PEM is very appropriate to detect a wide range of inversions, even with low coverage data. However, 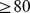
% of inversions located between segmental duplications are expected to go undetected by the most common sequencing strategies. In general, longer DNA libraries improve the detectability of inversions far better than increments of the coverage depth or the read length. Finally, we review the performance of three algorithms to detect inversions —SVDetect, GRIAL, and VariationHunter—, identify common pitfalls, and reveal important differences in their breakpoint precisions. These results stress the importance of the sequencing strategy for the detection of structural variants, especially inversions, and offer guidelines for the design of future genome sequencing projects.

## Introduction

In the last several years, genomic techniques have discovered an unprecedented degree of structural variation (SV) in multiple species, including humans [1]–[3]. This advance has spurred a renovated interest in the study of all kinds of SV, both in normal situations and in disease. Currently, one of the most used techniques for SV detection is paired-end mapping (PEM), which has been associated to high-throughput DNA sequencing methods [4]–[7]. Millions of pairs of short reads of DNA are sequenced from a DNA library of a target genome with a known length distribution. The two reads in a pair are the sequenced ends of a template molecule from the DNA library. When a pair of reads are mapped to a reference genome, they are expected to lay at a certain distance, and in a specific relative orientation. Deviations from these expectations are then interpreted as structural variations between the target and the reference genomes.

Several algorithms have been developed to translate the PEM data into a list of structural variants (reviewed in [8], [9]), and some studies have successfully applied them to whole human genomes [4], [10]–[14]. However, the proportions of false positives produced by PEM-based methods are high [4], [15], if not unknown [13], [16]. False negatives are also suspected to be many, especially in repetitive regions of the genome analysed [17]. The most common source of errors in PEM-based SV detection is probably mismapping, that is, the spurious alignment of reads to non-orthologous positions of the reference genome.

In principle, it is possible to analytically derive the probability of detecting a structural variant as a function of the sequencing strategy (e.g., template length, and sequencing effort; [18]), which could be used to estimate the likelihood of a candidate structural variant. However, these theoretical expectations are overly optimistic because they do not take into account the repetitive structure of the sequenced genome, nor the ambiguously mapped reads. A more realistic alternative is to use genome-specific simulations and empirical models of the SV-detection process. Recently, genome-specific simulations are being used to evaluate the performance of SV-detection software and to estimate rates of false positives and false negatives [19]–[21]. Most of such simulations lack a realistic distribution of sequencing errors, which is essential when researching mapping-related issues. Furthermore, simulated structural variation is either distributed randomly or copied from known variants. Neither strategy represents the real, unknown distribution of SV in the human genome. In particular, the tendency of SV to happen in repetitive regions is elusive for the most common detection methods, largely ignored by simulation studies, and overlooked in databases.

One particular type of SV that is especially problematic is chromosomal inversions, which simply change the orientation of a fragment of DNA. Polymorphic inversions have been studied in the species of *Drosophila* for decades [22], and they are also known to exist in human populations [23]. Interestingly, inversions could have important consequences on the genome both through the effect on nearby genes or the inhibition of recombination within the inverted region in heterozygotes. As such, they have been shown to be involved in phenotypic characteristics [24], susceptibility to genetic disorders [23], and evolution [25]. Traditionally inversions have been very difficult to detect and validate across a whole genome in a high-throughput manner, and are one of the less well characterized type of SV. PEM has the advantage of being able to discover balanced or dosage-invariant rearrangements, such as inversions and translocations, and has been used to predict several hundreds of inversions in different human individuals [4], [10]–[14]. Nevertheless, as mentioned above, very little is known about the proportion of inversions that are missed or incorrectly predicted by different mapping and sequencing methods, and current PEM predictions could be giving us an inaccurate and incomplete view of the inversions in the human genome.

**Figure 1:**
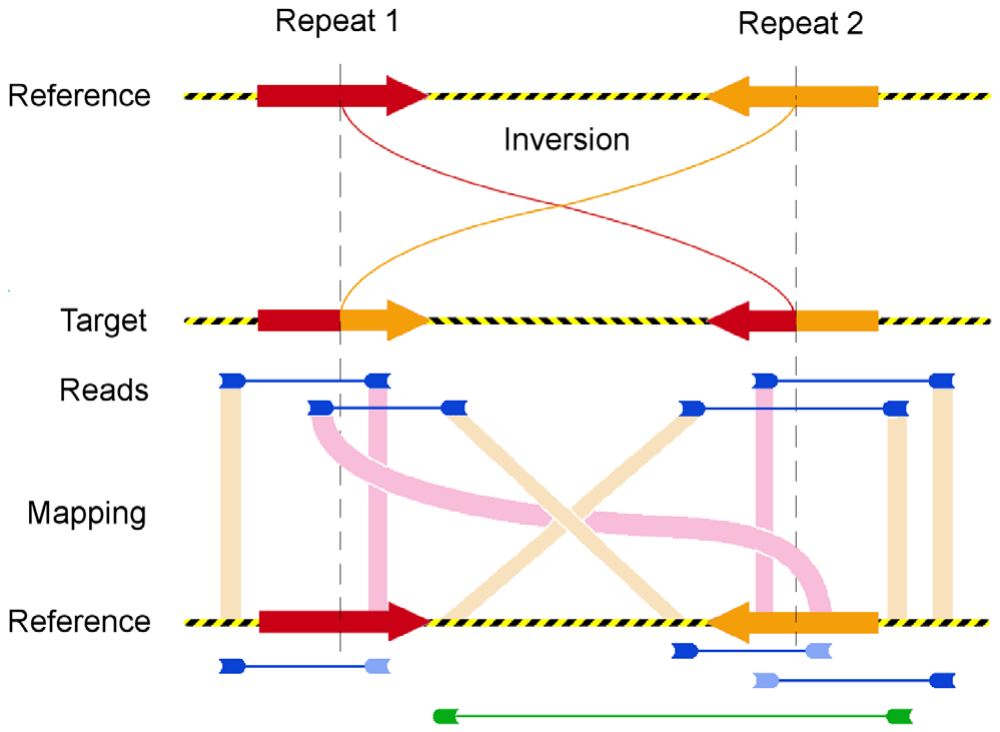
Inversion between the reference and the target genomes. The breakpoints (dashed lines) are located inside inverted repeats (red or orange arrows). Four pairs of reads that span the breakpoints are depicted in blue, with their sequenced ends in opposite orientations. Yellow bands indicate the correct mappings in the reference genome of ends located in unique sequences. The reads sequenced from a repeat are erroneously mapped to the alternative copy (pink bands), because concordant alignments are favored by the aligner. The mapped reads at the bottom are displayed in dark blue if correctly mapped or in light blue otherwise. The only discordant pair of reads that report the inversion is shown in green.

Inversions are expected to produce a very specific and distinct pattern of discordantly mapped reads, consisting on one of the ends being mapped in the unexpected orientation. Because this signature is known with absolute precision, in contrast with the expected distance between two mapped reads across an insertion or a deletion, inversions should be easier to detect by PEM methods. However, inversions are frequently located where it is most difficult to map reads uniquely. Inversions have been proposed to originate by two main types of mechanisms: non-homologous end-joining of random breaks in more or less simple sequences, or non-allelic homologous recombination between inverted repeats. Although each mechanism relative contribution to inversion generation is discussed and varies depending on the detection method [4], [10], [26], a big fraction of polymorphic inversions in humans, especially the largest ones (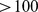 kb), are flanked by highly identical segmental duplications [23]. Therefore, many reads sequenced across inversion breakpoints are mapped concordantly (Figure 1), and the power of PEM methods to detect them is significantly reduced. As an example, the pilot study of the 1000 genomes project described several new big insertions and deletions in human populations, but neglected inversions because ‘methods capable of discovering inversions […] in low coverage data […] remain to be developed’ [27]. In a similar study, 80 individuals from natural populations of *Arabidopsis thaliana* were sequenced to a depth of 10–20 each with paired-end reads, but inversions were not reported yet [28].

In this study, we use computer simulations to estimate the sensitivity and the specificity of different sequencing strategies in detecting chromosomal inversions of different sizes and in different sequence contexts. We use human chromosome 1 as a model of the human genome, and we simulate realistic paired-end sequencing experiments with meaningful base qualities, sequencing errors, and sequence divergence between the target and the reference genomes. Simulated inversions are located either randomly or between inverted repeats, in order to represent two types of mechanisms of origin, either mediated by homology or not. We explain why the discovery of inversions from PEM experiments have had limited success, and make recommendations for future experiments. We also predict the expected levels of false positives and false negatives for each kind of inversion, under different strategies, and we compare the performance of three different SV-detecting algorithms: SVDetect [29], VariationHunter [16], and GRIAL (Martnez-Fundichely, S. Casillas, and M. Cáceres, unpublished data).

## Methods

### Simulation of inversions

From the reference sequence of human chromosome 1 (hg19), we simulated two target chromosomes: the *inversionless* chromosome, colinear to the reference, and the *inversionful* chromosome, including 948 inversions. Gaps in the reference genome (9.6% of its length) were substituted by random sequence of equivalent length and composition in both simulated chromosomes. We then introduced two main kinds of inversions: 424 randomly located inversions, and 524 inversions located between inverted repeats (Table 1). To generate the latter, we first identified all pairs of inverted repeats not more than 200 kb apart present in chromosome 1. We used three databases of such repeats: segmental duplications, self-alignments, and repeat-masked sequences, all downloaded from the UCSC Genome Browser ftp site. Inversions were distributed as evenly as possible between the three kinds of inverted repeats, making sure that they did not overlap and that they were separated by at least 50 kb. In all, 29 inversions were located between segmental duplications, 97 between other alignable regions, and 398 between repeat-masked fragments of the same type (Table 1). The distribution of inversion lengths is roughly linearly decreasing between 200 bp and 200 kb.

**Table 1:** Characteristics of the inversions simulated in four sequence contexts.

Sequence context	Number of inversions	Inversion size (bp)		Repeat length (bp)		Repeat identity (%)	
Random	424	61,756	(46,449)	NA		NA	
Repeat-masked	399	75,544	(57,549)	347	(383)	81.6	(7.3)
Alignable	98	16,716	(39,069)	201	(131)	94.2	(4.4)
Segmental dup.	29	102,469	(65,558)	19,962	(29,027)	89.2	(15.0)

Number and average size of inversions simulated in each type of sequence context, and average length and average percentage of identity between the two inverted copies flanking the breakpoints (repeat-masked and segmental duplications) or within their largest alignment blocks (alignable). The standard deviations are shown in parentheses.

The breakpoints of inversions between segmental duplications were located in the middle point of the longest tract of perfect identity between them, which is a good approximation of real inversions produced by non-allelic homologous recombination (M. Cáceres, unpublished data). To determine what was the longest tract of perfect identity between two copies, we performed either a global exhaustive alignment, if possible, or a local heuristic alignment with the program Exonerate and parsed the output. The identity between the two copies was recorded as the number of identical residues divided by the average length of the two copies. Similarly, the breakpoints of inversions situated between other alignable regions were chosen in the middle of the longest block of ungapped alignment between the two copies. The length of the chosen block and its percentage of identity were recorded. For inversions between masked repeats, breakpoints were located in the middle points of the copies. Their average length and the percentage of identity between them were also recorded.

### Sequencing

Because divergence between the sequenced and the reference genomes affects the ability to map the reads, we also introduced a non-trivial proportion of 0.005 mutations in the simulated copies of human chromosome 1 (hg19), including point mutations (80%) and indels (20%) of 1–4 bp. Several paired-end sequencing experiments of the two target genomes were simulated with the wgsim utility distributed with SAMtools [30]. To correct the homogeneity of sequencing errors along the reads produced by wgsim, we originally simulated the reads without any error, and then used a custom perl script to assign stochastic base qualities and add sequencing errors with a probability corresponding to the assigned quality. Base qualities were distributed along each read independently, according to a generalization of the empirical error models available in the package MetaSim [31].

We simulated three different read lengths that are representative of the available data in current and past paired-end genome sequences generated by the most popular sequencing technologies: 36, 75, and 150 bp. These read lengths were combined with five commonly used library template lengths from 250 bp to 40 kb (except when the template was shorter than twice the read length), generating 14 realistic sequencing experiments in each simulated chromosome (Table 2). Standard deviations were proportional to the template lengths, according to a linear regression estimated from empirical data from different types of real DNA libraries [4], [10], [27].

**Table 2:** Sequencing strategies and sequencing efforts of the inversionful chromosome.

Template (bp)	Read (bp)	Num. reads	Seq. depth	Phys. cov.
250 (15)	36	70,014,219	20.22	50.00
250 (15)	75	124,625,311	75.00	50.00
450 (27)	36	69,236,284	20.00	105.00
450 (27)	75	41,541,770	25.00	50.00
450 (27)	150	83,083,540	100.00	50.00
2,500 (150)	36	69,236,284	20.00	674.44
2,500 (150)	75	33,233,416	20.00	313.33
2,500 (150)	150	16,616,708	20.00	146.67
10,000 (599)	36	69,236,284	20.00	2,757.78
10,000 (599)	75	33,233,416	20.00	1,313.33
10,000 (599)	150	16,616,708	20.00	646.67
40,000 (2394)	36	69,236,284	20.00	11,091.11
40,000 (2394)	75	33,233,416	20.00	5,313.33
40,000 (2394)	150	16,616,708	20.00	2,646.67

Sequencing strategies tested, defined by the template and read lengths. Standard deviations of template lengths are shown in brackets. The number of reads simulated from the inversionful chromosome and their corresponding expected sequencing depth and physical coverage are shown.

When sequencing the inversionless chromosome, the number of simulated reads in each experiment was determined to generate an expected sequencing depth of 20. When sequencing the inversionful chromosome, though, we aimed at a sequencing depth of at least 20 and to a physical coverage of at least 50 (Table 2). We call ‘physical coverage’ what others have called ‘clonal coverage’ [18] or ‘span coverage’ [19], namely the number of times that a site lays between the two sequenced ends of a pair. We define the expected physical coverage as 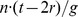, where 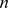 is the number of templates sequenced, 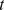 is the average template length, 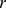 is the read length, and 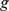 is the genome size. This assumes that the length needed to map the pair is not available to detect a breakpoint, which lets us focus on the problem of detecting inversions by different PEM strategies, and set aside the complementary approach of detecting them by the use of split reads (but see Discussion).

### Mapping

We used Novoalign (http://www.novocraft.com) to map the sequenced reads to the reference genome. We allowed for a score difference of 5 (default) between alternative alignments to consider the read ambiguously mapped, and we kept up to 100 alignments for each ambiguously mapped read. The alignments were done in paired-end mode, using the information of the expected distance between the two ends of a pair to find the most likely mapping and to determine if a pair is concordant or discordant. To favor concordant mappings over discordant ones, we set an SV penalty, which represents how much more likely a discordant mapping must be, relative to its concordant alternative, for it to be preferred (it is equivalent to the phred-scaled a priori probability of a breakpoint being covered by a read). Higher values of SV-penalty are expected to increase the specificity of SV detection and to reduce the sensitivity. We tested SV-penalty values between 0 and 70.

### SV-detection algorithms

Three SV-detection algorithms were used to identify common difficulties in the post-mapping stage of PEM data analysis: SVDetect [29], VariationHunter [16], and GRIAL (A. Martnez-Fundichely, S. Casillas, and M. Cáceres, unpublished data), which is available in http://grupsderecerca.uab.cat/cacereslab/grial. Care was taken to offer the same paired ends mapped in discordant orientation to all programs, while respecting their specific requirements. Because there is a trade off between template length and throughput of current sequencing technologies, we considered more realistic to downsample the reads from experiments with an expected physical coverage larger than 50 (see Table 2). Thus, we evened up the physical coverage, rather than the sequencing depth, across experiments before using the SV-detection algorithms.

SVDetect uses a sliding-window approach to first identify pairs of windows (links) connected by one or more discordant read. Redundant links are purged and reads within them are filtered. Finally, the program defines clusters of reads and identifies their corresponding structural variation. We set the minimum number of reads required to call a cluster to be 3, and followed the author's suggestion to set the lengths of both the window and its sliding step in order to be able to detect large SVs [29]. A mapping quality threshold of 20 was applied to the input reads, which proved to reduce the number of false positives significantly. GRIAL only predicts inversions. It relies on the average template length and on its standard deviation to apply some geometric rules and define a minimum range where the breakpoints must be (A. Martnez-Fundichely, S. Casillas, and M. Cáceres, unpublished data). As before, the minimum number of reads required to call a cluster was also 3, and a mapping quality threshold of 20 was used. Both GRIAL and SVDetect are hard-clustering algorithms, meaning that they assume a unique mapping for each read. In contrast, VariationHunter takes as input all possible mappings of each read, being aware of their mapping qualities. It applies a sophisticated algorithm to find the minimal set of compatible structural variants collectively supported by all discordant reads, so that each read only gives support to one variant [32]. Although VariationHunter usually work with all the potential read mappings provided by its companion aligner, MrFast [33], we instead parsed the SAM files produced by Novoalign into VariationHunter's native format, including only up to 100 alternative mappings for each read.

All three programs produce a set of chromosomal intervals where the breakpoints of the inversions are predicted to be. Predictions other than inversions (or ‘inverted segments’ and the like) were discarded. The length of the interval is the precision of each breakpoint prediction. If any program produced overlapping predictions, we merged them in one larger interval that included all of them. This was necessary for 90% of predictions across experiments by SVDetect, but infrequent for GRIAL (0.04%) or VariationHunter (0.2%). Then, the predictions were compared with the true locations of the breakpoints, and the numbers of true positives, false positives and false negatives were recorded for each program and sequencing strategy. The breakpoints predicted were also compared among programs.

In order to determine if false breakpoints were predicted on inverted repeats more often than expected, we counted the overlaps between false breakpoints and all the inverted repeats present in chromosome 1 (segmental duplications, repeat-masked regions, and other alignable segments). Then, we counted all the positions in the genome where a breakpoint prediction of certain length would have overlapped with one, two… or any number of repeats of each kind. From them, we determined the total expected number of overlaps that false breakpoint predictions by each program could have produced with each kind of repeat if they were randomly located. Finally, we used this number as the 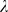 parameter of the Poisson distribution to test if the number of overlaps observed was higher or lower than the random expectation.

## Results

### Sequencing and mapping

Two simulated target genomes were generated: the inversionless and the inversionful (948 inversions of different types, see Table 1), derived from human chromosome 1 in the hg19 assembly. Each of them was paired-end sequenced 14 times, with different combinations of template and read lengths (Table 2). After sequencing, we mapped the reads to the reference genome using Novoalign. In all the experiments 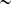
10% of the reads were not mappable, due to the presence of gaps in the reference sequence.

Because the original positions of the reads from the inversionless chromosome were known, we were able to measure their distances to the mapped positions and evaluate the true quality of the alignments. Table 3 shows some statistics of the performance of the aligner in the different experiments. We counted as correct all mappings within a distance to their expected position not larger than the length of the sequenced end, in order to account for potential deviations due to either small indels or alignment clipping. Between 1 and 3% of all mapped reads had at least one alternative mapping. In the majority of ambiguous mappings, the primary alignment is incorrect (Table 3), and the true alignment is to be found, if at all, among the secondary mappings.

**Table 3:** Summary statistics of the mapping of reads from the inversionless chromosome.

			Uniquely mapped (%)				Ambiguously mapped (%)		
			MAPQ 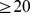		MAPQ 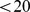		(all MAPQ 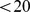 )		
Read (bp)	Template (bp)	Total simulated	correct	wrong	correct	wrong	correct	wrong	unmapped (%)
36	250	138,472,568	85.41	0.005	1.62	0.14	1.16	2.05	9.61
36	450	138,472,568	85.70	0.005	1.50	0.12	1.15	1.92	9.61
36	2,500	138,472,568	86.24	0.003	1.37	0.06	1.15	1.58	9.60
36	10,000	138,472,568	86.40	0.004	1.56	0.05	1.10	1.36	9.52
36	40,000	138,472,568	85.96	0.009	2.01	0.13	1.12	1.55	9.23
75	250	66,466,832	87.64	0.005	0.73	0.04	0.79	1.18	9.62
75	450	66,466,832	87.71	0.006	0.71	0.03	0.79	1.14	9.61
75	2,500	66,466,832	87.97	0.007	0.62	0.03	0.77	1.02	9.58
75	10,000	66,466,832	88.24	0.012	0.55	0.03	0.70	0.97	9.50
75	40,000	66,466,832	88.26	0.041	0.58	0.09	0.66	1.23	9.14
150	450	33,233,416	88.77	0.002	0.32	0.01	0.56	0.71	9.63
150	2,500	33,233,416	88.87	0.002	0.30	0.01	0.54	0.65	9.63
150	10,000	33,233,416	89.01	0.002	0.30	0.01	0.50	0.56	9.63
150	40,000	33,233,416	89.03	0.002	0.33	0.01	0.51	0.50	9.63

For each experiment, defined by the length of the reads and the average length of the templates, we show the total number of reads simulated from the inversionless chromosome and the percentages thereof that have been: mapped uniquely or ambiguously, with a mapping quality (MAPQ) of at least 20 or lower, correctly mapped or not, or unmapped. An ambiguous mapping is considered correct if the primary alignment is correct.

Both the template length and the length of the reads have positive effects on the mapping quality, with some nuances. It is remarkable that when the length of the read is shorter than 150 bp, templates of 40 kb produce more mapping errors than templates of 10 kb. This increase in the number of erroneously mapped reads is paralleled by a similar decrease in the number of unmapped reads. We interpret this as a result of the over-zealous alignment of unmappable reads, the presence of gaps in the reference genome, and the proportionality between the average template length and its standard deviation (Table 2). Short reads proceeding from regions not represented in the reference genome are more likely to have spurious concordant alignments when the length of the template is known with less precision.

The application of a mapping quality threshold of 20 (mapping error probability 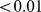), reduces the mapping error rate by about 2 orders of magnitude. Such an improvement in average mapping quality comes at the cost of removing more well mapped reads than erroneously mapped ones. Remarkably, all ambiguously mapped reads, that is, all reads with at least two possible mappings within 5 score points from each other, are removed by this filter.

### Mapping specificity in inversion detection

Because we simulated Illumina reads, expected to map in forward-reverse orientation, only reads with forward-forward or reverse-reverse orientations are informative of the presence of inversions. These discordant orientations may also arise from mismapping. We used the inversionless chromosome to determine the probability of finding spurious inversion-like orientations in paired-end mappings to human chromosome 1, using different combinations of template length and structural variation (SV) penalty (see Methods).

If ends are mapped independently of each other (that is, with a null SV penalty), about 2% of all pairs with 36 bp reads are mapped in discordant orientations, suggesting the spurious presence of inversions (data not shown). A positive SV penalty rapidly decreases this proportion, which asymptotically approaches 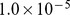 by SV-penalty 70. Different template lengths do not significantly change this figure. Longer reads were mapped only with an SV-penalty of 70.

Most of the discordant paired ends from the inversionless chromosome are assigned a low mapping quality. If reads are 36 bp long, and only paired ends having both a mapping quality of at least 20 are considered, around 95% of the orientation-discordant mappings are removed, while only 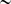
5% of concordant reads are affected by the filter. The effect of the mapping quality threshold is equivalent for all values of SV penalty and template length tested (data not shown). Overall, with 36 bp reads, the combination of a mapping quality 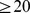 and an SV penalty of 70 reduces the frequency of false orientation discordant paired-ends about 5 orders of magnitude, to between 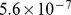 and 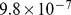. Longer reads from the inversionless chromosome, with a mapping quality of at least 20 (and mapped with an SV-penalty of 70), include proportions of orientation-discordant pairs always lower than 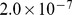. If reads from the inversionful chromosome have similar rates of mismapping, from a physical coverage of 50 we expect between less than 1 (reads longer than 36 bp and templates longer than 250 bp) and 39 (36 bp reads, 250 bp templates) spurious orientation-discordant pairs with a mapping quality of at least 20, that would suggest the presence of false inversions.

### Mapping sensitivity in inversion detection

To determine the ability of PEM experiments to detect inversions, we computationally sequenced the inversionful chromosome using different combinations of read and template lengths (Table 2). Before the application of any SV-detection software, we determined the performance of the alignment software at providing evidence of the breakpoints. We applied an SV penalty of 70, necessary to remove most false positives (see above). A pair of ends sequenced from alternative sides of a breakpoint is potentially informative of the existence of the breakpoint. For every breakpoint, we counted the potentially informative pairs obtained with each sequencing strategy, and how many of them were mapped correctly, erroneously mapped and unmapped.

The informative physical coverage of a breakpoint depends on the sequencing strategy and on the length of the inversion. The expected informative physical coverage can be expressed as the product of the total number of templates sequenced and the probability that a template encompasses a single breakpoint between its two sequenced ends. Assuming that, as it is the case in our experiments, average template lengths are larger than twice the read length, and inversions are larger than the reads, then:
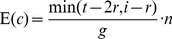
(1)where 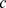 is the physical coverage, 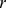 is the length of the reads, 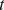 is the average length of the templates, 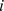 is the length of the inversion, 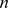 is the sequencing effort in number of templates sequenced, and 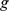 is the length of the genome.

**Figure 2:**
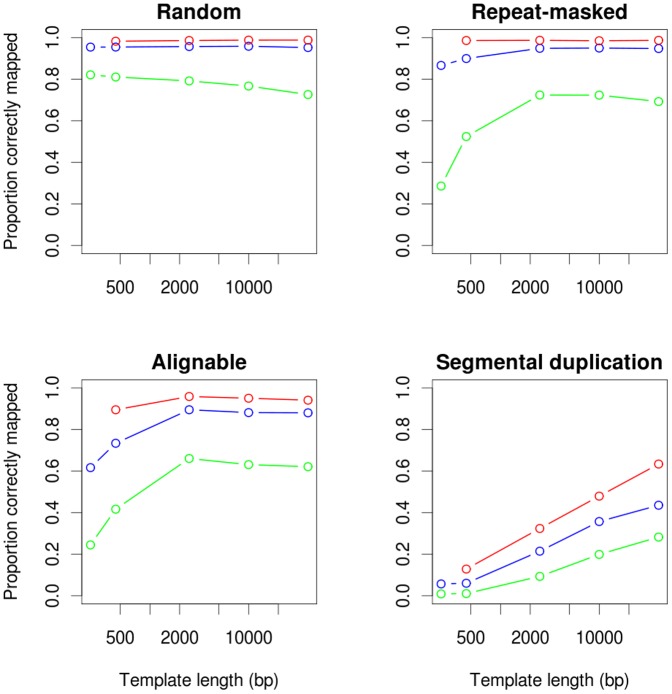
Average portion of potentially informative reads that are correctly mapped across a breakpoint. Informative reads are represented as a function of the template length for inversions located in four different sequence contexts. Colors represent the three read lengths: green, 36 bp; blue, 75 bp; and red, 150 bp.

Equation 1 describes well the number of reads actually sequenced across breakpoints. However, a variable portion of those reads are either unmapped or, more often, erroneously mapped. Figure 2 shows the average proportion of pairs of reads sequenced across a breakpoint that are correctly mapped in each experiment for inversions located in 4 different contexts: 1) randomly, 2) between inverted repeat-masked sequences, 3) between other inverted alignable regions, and 4) between inverted segmental duplications. The rest are erroneously mapped elsewhere, many as concordant (a small, and rather constant fraction of unmapped reads are not counted there).

As shown in Figure 2, reads of 36 bp (green points) with templates 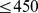 bp perform quite badly for all inversion types and have a probability of being well mapped around inverted repeats well below 0.6. It can also be seen that long templates are instrumental to correctly map reads across repeats. While short repeats, such as those identified by RepeatMasker and other alignable regions, are effectively bypassed with 2500 bp-long templates, most segmental duplications are challenging even for 40 kb-long templates.

**Figure 3:**
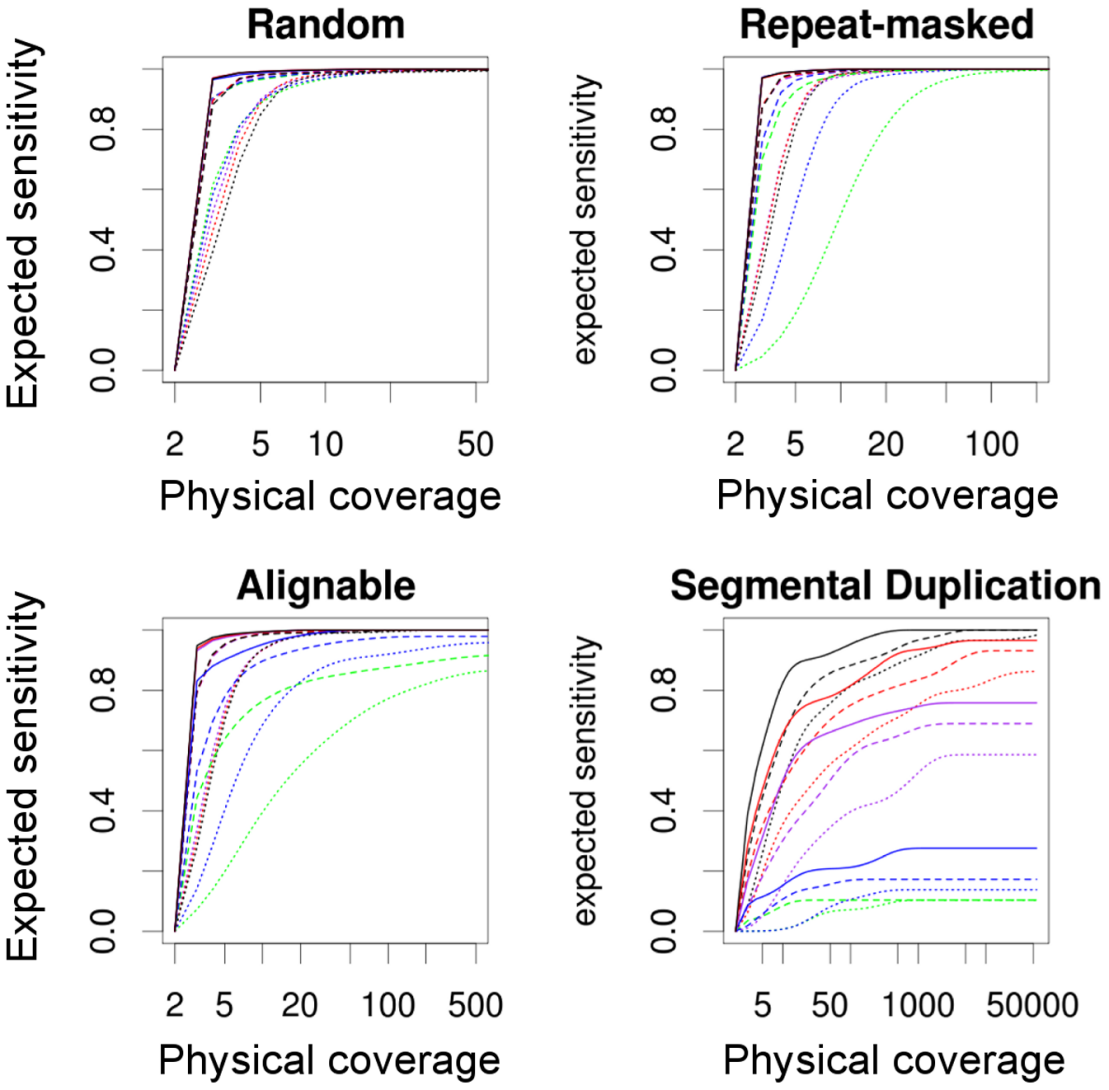
Relationship between physical coverage and the expected sensitivity of different sequencing strategies to detect inversions. The expected sensitivity is based on the probability of correctly mapping paired ends across inversion breakpoints in four different sequence contexts. Inversions are assumed to be longer than the templates. The sequencing strategy is defined by the read length: dotted lines, 36 bp; dashed lines, 75 bp; solid lines, 150 bp; and by the template length: green, 250 bp; blue, 450 bp; purple, 2.5 kb; red, 10 kb; and black, 40 kb. Notice the different ranges of physical coverage among plots.

The values shown in Figure 2 are averages across inversions. For each inversion, we estimated the probability of mapping a pair of ends correctly across one of its breakpoints, if at least 50 pairs had been simulated covering its breakpoints. These probabilities where then used to calculate the expected number of breakpoints detected by at least 2 paired ends with a given physical coverage and a given sequencing strategy (Figure 3). Our results show that low physical coverages can detect very efficiently randomly generated inversions. However, for inversions located between inverted repeats, maximal inversion detection requires quite different amounts of physical coverage depending on the PEM conditions, and suboptimal sequencing strategies are predicted to fail to detect a substantial amount of inversions located between segmental duplications, irrespectively of the sequencing effort. In particular, a physical coverage of 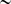
50 can achieve sensitivities higher than 90% in all sequencing contexts, with average template length of 40 kb and reads of 150 bp. Notice that such an experiment would produce a sequence coverage (i.e. sequencing depth) of only 0.4. In addition, easier to obtain libraries of 2.5 kb perform also very well for most types of inversions, except those mediated by segmental duplications.

**Figure 4:**
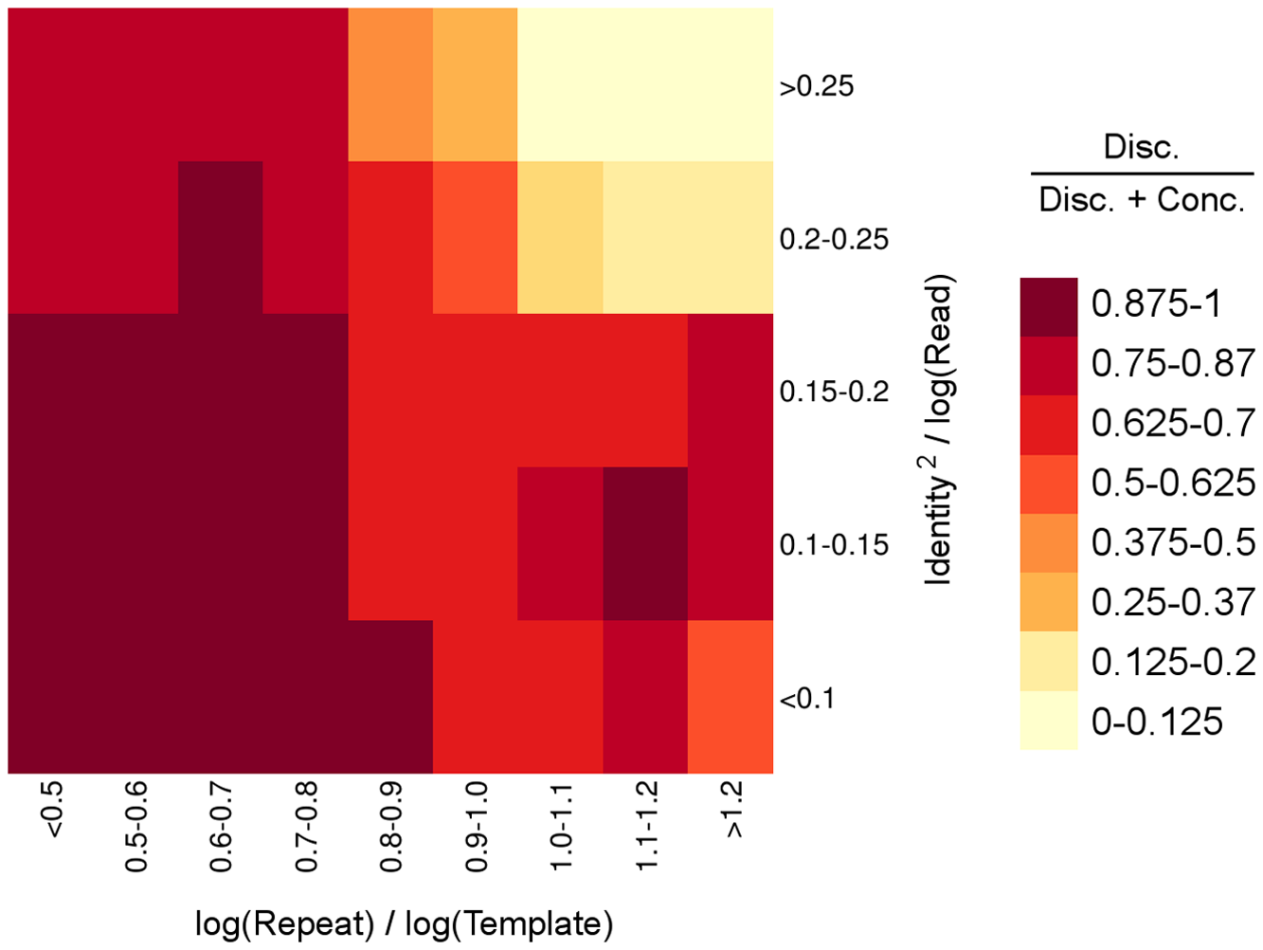
Average proportion of pairs of reads mapped across a breakpoint that are correctly mapped as discordant. Correct discordant read pairs are expressed relative to all reads mapped across the same breakpoint, as a function of the length of the repeat (relative to the length of the template) and the identity between the copies (relative to the read length). Data from all 14 simulated paired-end sequencing experiments are used. Cells may have different standard errors, due to differences in the total number of reads used to calculate the proportion of discordant pairs in each situation.

The probability of correctly mapping a pair of reads across a breakpoint is expected to depend on the characteristics of the inverted repeats present around the breakpoints, if any. At least, the length of the repeats and the similarity between them must affect directly the fraction of templates spanning a breakpoint that are mapped either discordantly or concordantly across the breakpoint. The expected value of that proportion (discordant over the sum of concordant and discordant) in a candidate breakpoint would be useful to determine the likelihood of that candidate. Thus, we attempted to fit a generalized linear model of the proportion of templates sequenced across a breakpoint that are mapped across that breakpoint either concordantly or discordantly, using characteristics of the inversion and of the sequencing strategy as predictors. We failed to correct the overdispersion present in all the models that we tested. We suspect that the specific distribution of mismatches along the alignment of the two inverted copies, and the amount and distribution of gaps thereof, which were not characterized, significantly affect the chances of a read being mapped to the correct copy. In any case, we captured part of the pattern of variation with two compound variables (interactions) using the data from the 14 experiments (Figure 4). First, the interaction between the length of the repeat and the length of the template is apparent in Figure 1: only templates longer than the repeats may have ends with unique sequences, that can be correctly mapped. And second, the interaction between inverted copies identity and the read length represents that longer reads are more likely to contain a difference between repeat copies than shorter reads from the same repeat. The logarithmic transformation of all lengths and the squaring of the identity improved the quality of the relationship.

### Sensitivity, specificity and precision of SV-detecting algorithms

**Figure 5:**
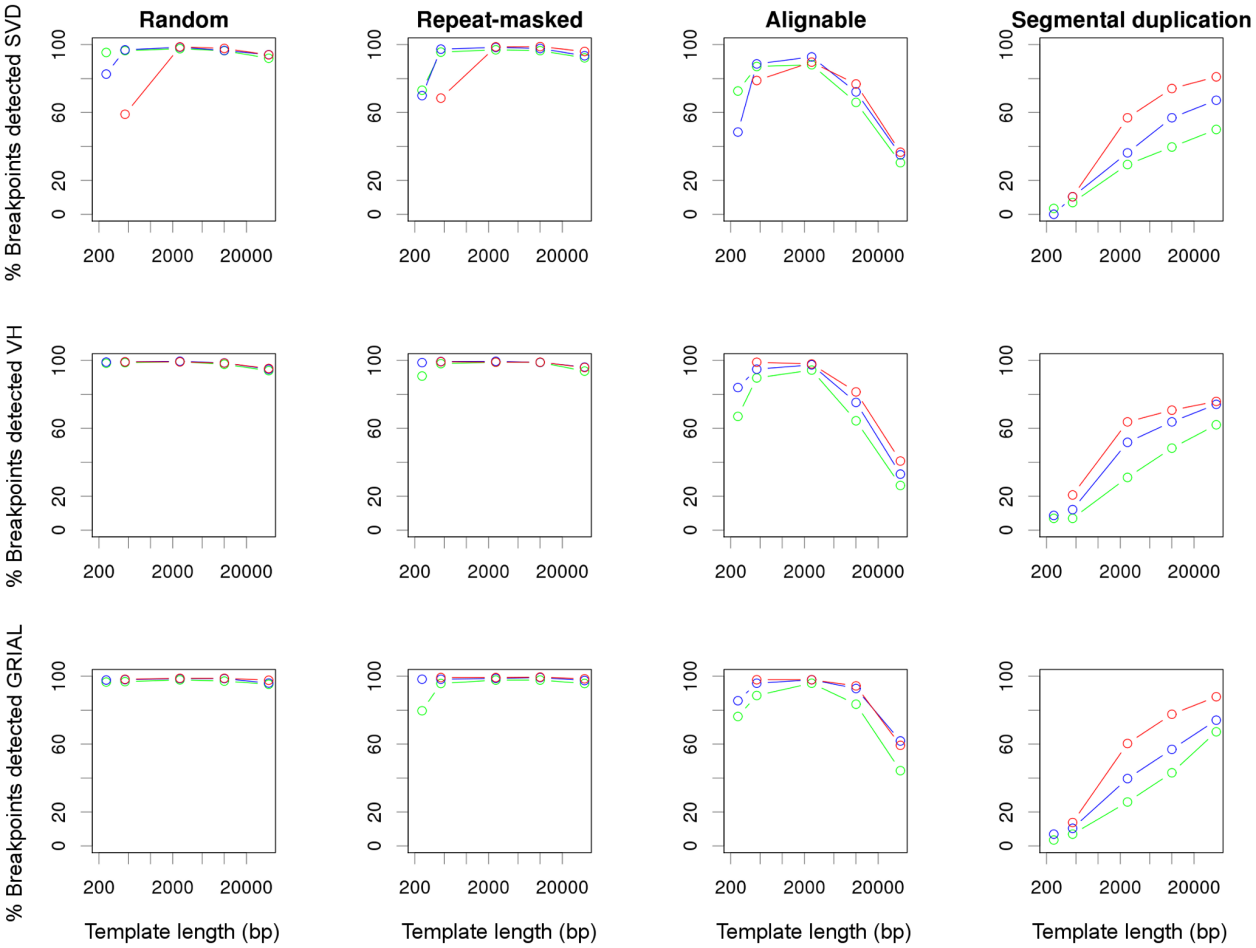
Percentage of inversion breakpoints from each sequence context that are successfully detected by different programs. Results from SVDetect (SVD, upper row), VariationHunter (VH, middle row), or GRIAL (bottom row) are plotted against the template length used. Colors correspond to the length of the reads: green, 36 bp; blue, 75 bp; and red, 150 bp.

The post-mapping analysis of PEM data to discover inversions may introduce its own biases. We used three different algorithms designed to detect inversions and other SV from PEM data to identify common sources of false positives and false negatives: SVDetect [29], VariationHunter [16], and GRIAL (A. Martnez-Fundichely, S. Casillas, and M. Cáceres, unpublished data). Figure 5 represents the percentage of breakpoints of each type of inversion detected by each program with different sequencing strategies. In this comparison, the physical coverage was kept at 50 across experiments, that is, longer templates entail fewer paired ends sequenced (see Methods).

The sensitivity (proportion of true breakpoints correctly predicted) of the three algorithms (Figure 5) is in general close to, but sometimes lower than, what expected from the mappability of the reads around breakpoints (Figure 3, for a physical coverage of 50). As predicted by Figure 4, sensitivity of shorter template libraries decreases with highly identical inverted repeats, especially segmental duplications. In addition, templates of 40 kb recall fewer breakpoints than 10 kb templates in most sequence contexts. This is due to a higher proportion of inversions being shorter than the template, and therefore receiving lower useful physical coverage (see Equation 1), than if sequenced with shorter templates. This effect is very pronounced in the case of inversions located between alignable regions, because they are on average the shortest (Table 1). If the number of paired ends sequenced or the sequencing depth, instead of the physical coverage, was kept constant across experiments, longer templates would always outperform shorter ones, as suggested by Figure 3 (data not shown).

Inversion detection for random and repeat-masked inversions with VariationHunter and GRIAL is almost 100%. In contrast, SVDetect does not reach the same sensitivity with short templates (250 and 450 bp). A careful inspection of these cases showed that SVDetect is calling inversion breakpoints some base pairs off the true breakpoints, thus producing false positives (see below). On the other hand, using 150 bp reads and 40 kb templates, GRIAL detected 51/58 breakpoints within segmental duplications, while SVDetect and VariationHunter detected 47/58 and 44/58, respectively. According to Figure 3, 53.5 breakpoints were expected to be detectable, on the bases of the mappability of the reads. Across experiments, GRIAL was about 6% more sensitive than SVDetect, and 0.4% more sensitive than VariationHunter.

**Figure 6:**
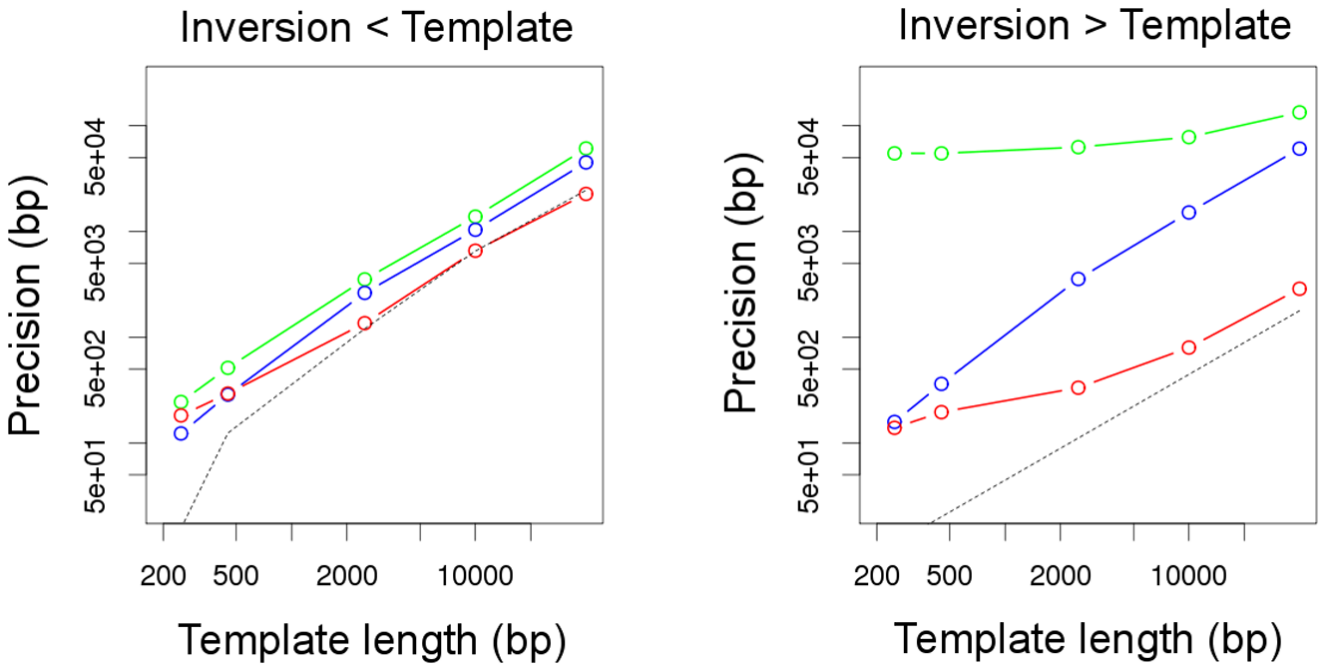
Precision of breakpoint prediction plotted against the length of the template. The average size of the predicted range of a breakpoint is represented separately for inversions smaller (left) or larger (right) than the template. Colors correspond to the programs used to predict the breakpoints: green, VariationHunter; blue, SVDetect; and red, GRIAL. The dashed lines correspond to the theoretical expected precisions, obtained either from equation 3 in reference [18] for large inversions, or from the average difference between the inversion size and the template length for small inversions.

We also measured the average precision attained by each program in their breakpoint predictions. In all cases, the ranges of positions where breakpoints are predicted to lay are larger than the theoretical expectation derived by Bashir et al. [18] for large inversions, assumed to be randomly located (Figure 6). As expected, the breakpoints of inversions smaller than the template length cannot be detected with a precision better than the difference between the length of the template and the length of the inversion. This reflects the fact that for a pair of ends to be informative, the read sequenced from outside of a small inversion must be at a certain distance, such that its partner is sequenced from inside the inversion. For both size classes, but in particular for big inversions, GRIAL achieves finer precision than the other two programs, and VariationHunter offers the coarsest precision. Note that both axes in Figure 6) are in logarithmic scale to appreciate how substantial the differences are.

**Figure 7:**
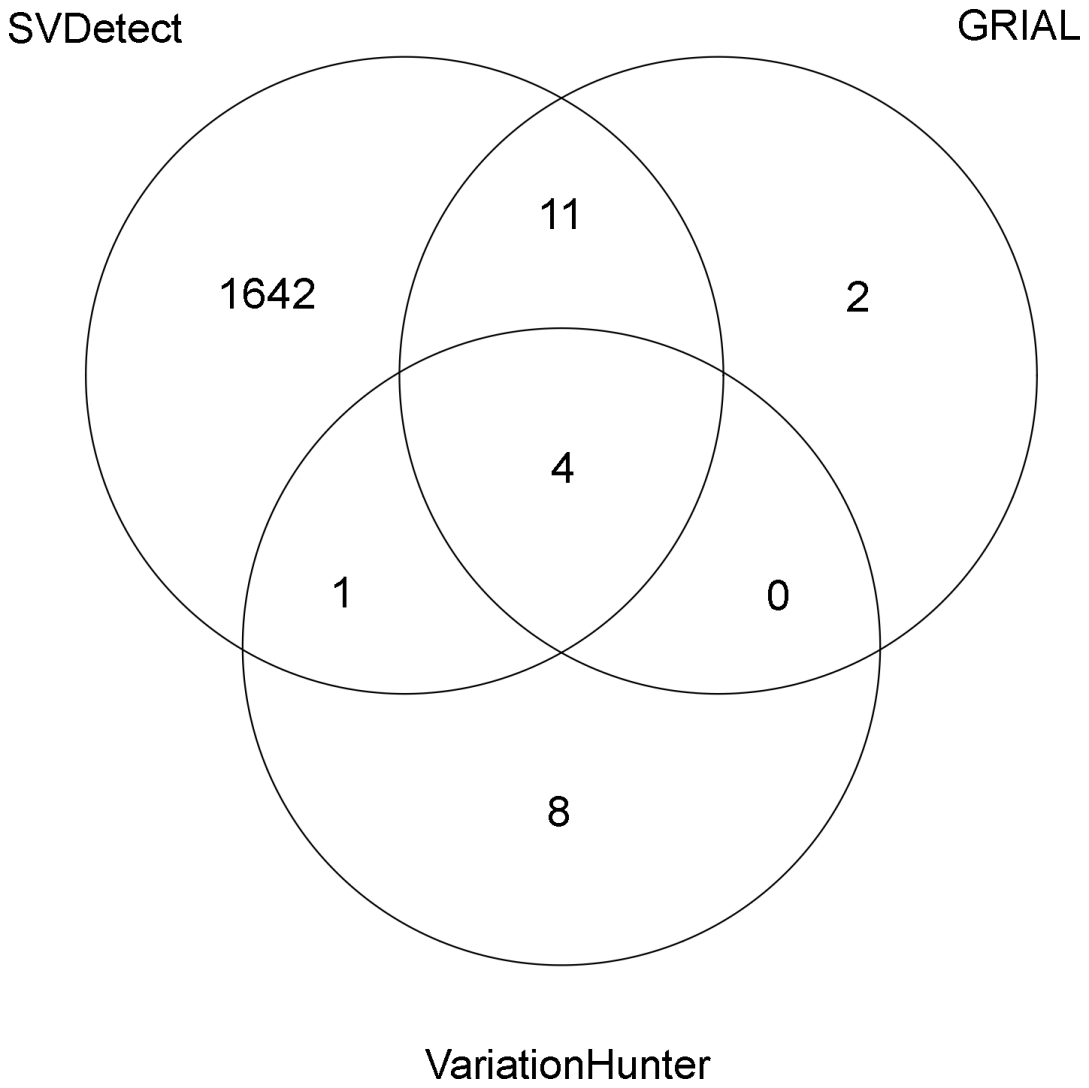
Comparison of the false breakpoints predicted by the three programs. Templates of 250 bp and 75 bp reads were used. The sharing of a breakpoint between two programs imply that their predictions overlap in at least one base and are of the same kind, namely, either the first or the second breakpoint of an inversion.

In addition to the true positives shown, all three programs produce a number of false positives (Table 4), which in general are higher for short templates. We compared the positions spanned by the false predictions among the three programs (Figure 7). The false positives predicted by VariationHunter tend to be different from those predicted by either GRIAL or SVDetect. We attribute these differences to the fact that VariationHunter uses ambiguously mapped reads with low mapping quality, that neither GRIAL nor SVDetect use.

**Table 4:** False inversion breakpoints called by three SV-detection algorithms.

Read (bp)	Template(bp)	SVD	VH	GRIAL
36	250	67 (0.0418)	11 (0.0065)	10 (0.0062)
36	450	6 (0.0034)	10 (0.0055)	4 (0.0023)
36	2,500	1 (0.0006)	5 (0.0027)	0 (0.0000)
36	10,000	0 (0.0000)	3 (0.0017)	0 (0.0000)
36	40,000	1 (0.0006)	2 (0.0012)	0 (0.0000)
75	250	1,658 (0.5508)	13 (0.0072)	17 (0.0095)
75	450	215 (0.1080)	7 (0.0038)	4 (0.0022)
75	2,500	6 (0.0033)	3 (0.0016)	0 (0.0000)
75	10,000	5 (0.0028)	4 (0.0022)	0 (0.0000)
75	40,000	0 (0.0000)	6 (0.0036)	0 (0.0000)
150	450	1,527 (0.5591)	10 (0.0054)	13 (0.0071)
150	2,500	15 (0.0081)	12 (0.0064)	0 (0.0000)
150	10,000	17 (0.0093)	8 (0.0044)	1 (0.0005)
150	40,000	4 (0.0024)	8 (0.0047)	1 (0.0006)

Number of false inversion breakpoints predicted by SVDetect (SVD), VariationHunter (VH) or GRIAL under each sequencing strategy, defined by the template and the read lengths. In parentheses, the proportion that these false positives represent among all the predictions.

We carefully looked at the origin of false positives and distinguished three different types. First, a small number of reads originally colinear with the reference (no more than 50 per experiment) were erroneously mapped in discordant orientation and gave support to false breakpoints, at least in SVDetect and in GRIAL (we could not keep track of what reads supported each prediction from VariationHunter). This is in agreement with the small number of false positives expected from erroneous mappings (see section on the Mapping specificity in inversion detection). We do not observe this kind of false positives when the template lengths are at least 2500 bp long or if the reads are at least 150 bp long. In principle, an SV-penalty higher than 70 during mapping could also reduce the number of this kind of false positives (not tested).

Second, truly discordant reads, originated across true breakpoints and mapped in the correct (discordant) orientation, but to an erroneous location, gave rise to false predictions. In the experiment with reads of 75 bp and templates of 250 bp, GRIAL predicted 10 false inversions (involving 17 false breakpoints, and 3 true breakpoints assigned to wrong inversions) and 7 of them are also predicted by SVDetect. These common false inversions are due to mismapped reads, and they are larger than 20 Mb. In contrast, VariationHunter filters out inversion predictions larger than 1 Mb, although it does predict individual, unpaired breakpoints in other locations.

And third, there are correctly mapped reads that are not well interpreted by the SV-detection algorithm, and give rise to ‘false’ breakpoints that do not overlap true breakpoints, but lay close. Across experiments, 88% of SVDetect's false breakpoints (see Table 4 and Figure 7) lay within 50 bp of a true breakpoint, and they predict inversions that do overlap with real inversions. Also the two false positives predicted by GRIAL with reads of 150 bp and template lengths of 10 kb and 40 kb are very close (at 23 and 62 bp, respectively) to real breakpoints. These two false positives may be due to random departures from the expected template lengths, upon which GRIAL predictions heavily depend (A. Martnez-Fundichely, S. Casillas, and M. Cáceres, unpublished data).

Just as inverted repeats are hotspots of false negatives (see section on Mapping sensitivity in inversion detection), they can also generate false positives, due to the possibility of mapping reads to either copy. To understand better the origin of false positives, we compared their positions with those of all the segmental duplications, RepeatMasker-filtered regions and other alignable regions present in chromosome 1. In all the experiments with average template lengths of 250 or 450, the false breakpoints predicted by GRIAL or by SVDetect overlap with either repeat-masked regions or with other alignable regions more often than expected by chance; but they did not overlap segmental duplications more frequently than expected (data not shown). In contrast, the false positives predicted by VariationHunter in 4 experiments did overlap segmental duplications more often than expected by chance (at 0.01 significance level, Poisson test), and they also overlapped other alignable regions (not repeat-masked segments) more often than expected by chance in some experiments.

## Discussion

Currently there is a great interest in the complete characterization of SV at a genome level, with multiple projects to sequence whole genomes using different PEM strategies. However, it is not clear to what extent these projects are giving us an adequate picture of the SV present in the human genome. Therefore, it is important to have quantitative estimates as realistic as possible of the amount of variants that we may be missing or describing incorrectly.

The mapping stage of PEM data analysis caps the sensitivity of any SV-detection program. For well understood reasons, inversions between segmental duplications may be undetectable, under some experimental designs. Unfortunately, most PEM experiments performed to date were done with very small templates (e.g., [11], [12], [27]; but see [10]) that are not suited to detect inversions between inverted repeats (Figure 5). Around 90% of the paired-end sequencing experiments (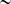
80% of the reads) generated by the 1000 genomes project have template lengths below 500 bp (according to the sequence indexes downloaded from their ftp site on October 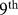, 2012). These template lengths, combined with modest coverages, are expected to miss more than 80% of the inversions between segmental duplications and around 5–50% of the inversions between repeat-masked or other alignable regions. Neither an increase in coverage, nor an improvement in SV-detection algorithms can prevent false negatives completely. It is also important to note that our sensitivity estimates may be overly optimistic, since we have simulated inversions between inverted repeats with identities as low as 60%, whereas real inversions are probably enriched in highly identical repeats. Thus, PEM studies have been systematically missing most of the inversions present between inverted repeats, and a similar problem may affect other types of structural variants. The actual relative abundance of inversions between inverted repeats is impossible to evaluate with current data from massively parallel paired-end sequencing studies, precisely due to the ascertainment bias against them.

It is known that longer templates improve the assembly in *de novo* sequencing projects [34], and extend the range of insertions that can be discovered by PEM [8]. However, little emphasis has been put on the importance of template length for inversion discovery. When detecting inversions, longer templates always improve sensitivity (Figure 2) and specificity (Table 4). If longer templates were used, the bias against inversions between inverted repeats could be traded for a bias against short inversions, but only as long as current technologies impose a trade off between the template length and the throughput. When designing an experiment, one could give priority to inversions between inverted repeats, and choose first the longest average template length available and then, the affordable sequencing effort. For example, Kidd et al. [10] used average template lengths of 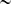
40 kb (fosmid genomic libraries), and sequenced about 400 bp of each end. They reached a sequencing depth of about 0.3 per individual, which, according to Equation 1, implies that the breakpoints of inversions shorter than 3 kb were expected to be physically covered less than once. In addition, Bashir et al. [18] reported a trade-off between detectability (template length) and breakpoint precision for large inversions in random locations, and they recommended a mixture of long and short template lengths to optimize both. Although this trade-off progressively vanishes with increasing physical coverage, it has an important corollary: the longer the templates, the higher the proportion of inversions that are shorter than the templates. In Figure 6, it can be seen that for a physical coverage of 50 the loss of precision due to longer templates in large inversions is small compared to that in inversions shorter than the templates. Thus, a coarse precision may be the price to pay for the detection of inversions between inverted repeats.

From our results (figures 2, 4, and 5), it is apparent that both longer reads and longer templates improve inversion detectability. In most of the genome, sequenced ends of 150 bp perform almost as well as possible. The constant development of sequencing technologies offers ever longer reads, going up to several kilobases in the case of Pacific Biosciences or Illumina's Moleculo technology. Eventually, long enough reads with high enough quality could override the need for paired-ends, and inversions would be detected by direct sequencing. However, increasingly longer reads will not help much for the detection of inversions located between large segmental duplications, but longer DNA libraries would. The breakpoints of an inversion located between inverted repeats are virtually invisible at the sequence level within the repeat, what renders split reads useless in this context. Longer sequences could be useful to map reads more accurately in the two inverted repeats, although in highly identical regions the mapping would rely at most in a few base differences between copies. These differences are known to vary between individual genomes and make the mappings that are not based in unique or quite divergent sequences unreliable.

In terms of sensitivity, the three programs tested perform similarly (Figure 5), stressing the importance of the sequencing design and the mapping stage. However, the programs differ significantly in terms of precision, GRIAL being the program with the most accurate breakpoints (Figure 6). In terms of false positives, the apparently high false discovery rate by SVDetect (Table 4, and Figure 7) is mostly due to the predictions missing the actual breakpoints by a few base pairs. VariationHunter is the only tested algorithm producing an excess of false positives in segmental duplications, that we attribute to its usage of low quality, secondary mappings. To avoid those false positives, either the discordantly mapped reads must be further filtered, or their mapping qualities should be more accurate.

Even low rates of false positives can produce high posterior error probabilities if inversions are rare [35]. On the other hand, if inversions (and maybe other kinds of SV) are frequent between the target and the reference genomes, as in the case of cancer genomes, the rate of false positives could be even higher. High levels of SV could produce large numbers of false positives because pairs of reads that span a breakpoint have a higher chance of being mapped to a wrong location *and* in a discordant orientation than those colinear to the reference. Thus, part of the false positives predicted here are due to pairs of reads sequenced across true breakpoints and mismapped, and they could be considered an artifact of the high density of the simulated inversions. Our results suggest that false positives can be kept low by using a stringent SV-penalty during mapping, filtering out low quality reads, choosing an appropriate algorithm, and using templates of at least 2.5 kb (Table 4). However, our simulations represent a best-case scenario, any departure from which will make it more difficult to detect the true inversions and to avoid the false ones. For example, the presence of other types of SV, and especially the presence of complex rearrangements, are expected to increase the rate of false positives, as mentioned earlier.

Polymorphic inversions are more likely to be detected where they are less likely to happen, namely in non-repetitive sequences; and difficult to detect where they are more likely to be, that is, between inverted repeats (Figure 5). As a result, the frequency of polymorphic inversions in the human genome could be underestimated in one hand, and overestimated due to false positives in the other. Supposedly simple tasks such as comparing the frequency of inversions among chromosomes, or estimating the total number of inversions in one genome, are not supported by any SV-detection algorithm to date, because the unknown numbers of false positives and false negatives would bias the results. Yet, with the information contained in PEM data, it should be easier to estimate the total number of inversions, than to enumerate all of them. Thus, we think that SV-detection algorithms will keep evolving to implement sound statistical models with estimates of both false positives and false negatives.

One step in this direction is the recent appearance of GASVPro, an SV-detection algorithm that implements a probabilistic model to determine the most likely set of structural variants supported by PEM data from one individual [36]. GASVPro uses multiple possible alignments of discordant reads, and approximates the posterior probabilities of the mappings. Although GASVPro does not explicitly estimate the total number of SVs, nor it reports the probability that a prediction is false, its probabilistic formulation would allow such extensions. Instead, GASVPro follows the trend of reporting a list of variants, biased as it may be. Therefore, it is not surprising that even GASVPro has a very low rate of recall of known inversions from two sequenced individuals, and apparently high rates of false positives, just as all other programs tested by the authors (Tables 2 and 3 in [36]). Other recent developments in SV-detection algorithms tend to use evidence from both paired-ends and split reads to improve the definition of breakpoints [37], [38]. These methods take the most of the data at hand and improve the sensitivity and the specificity in some circumstances. However, they fail to address the main concern raised by our results, namely the overlooking of inversions between inverted repeats, where split reads do not add any information.

In summary, current SV-detection algorithms fail to account for the heterogeneous distribution of SV, and in particular of inversions, along the genome; and they fail to account for the also heterogeneous probability of false positives. In order to study their mechanisms of origin and to perform population genetic analyses of inversions, we need to estimate parameters of an explicit model of SV distribution, rather than an incomplete and biased list of differences between two genomes, and they will have to pay attention to the genome-specific repetitive structure. Future improvements of both algorithms and sequencing strategies are expected to give us a better idea of the genomic landscape of SVs in general, and inversions in particular.
